# Roflumilast restores cAMP/PKA/CREB signaling axis for FtMt-mediated tumor inhibition of ovarian cancer

**DOI:** 10.18632/oncotarget.22866

**Published:** 2017-12-02

**Authors:** Shipeng Gong, Yongning Chen, Fanliang Meng, Yadi Zhang, Huan Wu, Fei Wu

**Affiliations:** ^1^ Department of Obstetrics and Gynecology, Nanfang Hospital, Southern Medical University, Guangzhou, Guangdong 510515, P.R. China; ^2^ Department of Obstetrics and Gynecology, The Second Affiliated Hospital, Chongqing University of Medical Sciences, Chongqing 400010, P.R. China

**Keywords:** roflumilast, cAMP, CREB, FtMt, ovarian cancer

## Abstract

The abrogation of cAMP generation by overexpression of PDE isoforms promotes the inflammatory pathology, and the PDE inhibitors have showed the potential anti-inflammation effects in clinical. However, the function of PDE inhibitors in cancer treatment remains unclear. We here investigated the role of PDE4 inhibitor Roflumilast in the treatment of ovarian cancer. We found that Roflumilast could effectively inhibit the proliferation, and induce apoptosis and cell cycle arrest in two ovarian cancer cell lines OVCAR3 and SKOV3. Meanwhile, the cAMP/PKA/CREB signals was activated by Roflumilast, which was accompanied by the up-regulation of mitochondrial ferritin (FtMt) level. Interestingly, forced expression of FtMt in ovarian cancer enhanced the apoptosis and inhibited tumor growth and the PKA inhibitor H89 and knockdown of CREB significantly repressed the expression of FtMt to restore the tumor proliferation and inhibit apoptosis. In addition, we found that Roflumilast-induced phosphorylated CREB directly promoted transcription of FtMt, indicating that Roflumilast up-regulated the expression of FtMt in ovarian cancer via cAMP/PKA/CREB signals. The anti-tumor role of Roflumilast *in vivo* was also demonstrated, the treatment of roflumilast effectively inhibited tumor proliferation and elevated the FtMt expression to restrict the tumor growth via the activation of cAMP/PKA/CREB signals in ovarian cancer.

## INTRODUCTION

As the most lethal gynecologic cancer, ovarian cancer is the leading cause of gynecologic cancer death in developed countries, with an incidence of 6.1 cases per 100.000 women, a rate of mortality of 4.3 deaths per 100.000 women [1, 2]. Once the patients develop recurrent disease, standard therapies (debulking surgery and platinum-based chemotherapy) will be unsuitable for those patients, leading to the poor prognosis of ovarian cancer patients [3]. Therefore, understanding ovarian cancer pathogenesis and the mechanism of its growth, metastasis and recurrence is critical to identify new targets or alternative strategy for the treatment of ovarian cancer.

Cyclic adenosine monophosphate (cAMP) as one of the most ancient signaling molecules has been found to convert the extracellular signals into specific cellular responses to activate protein kinase A (PKA) and cyclic AMP response element-binding protein (CREB) etc., which involved into a wide range of cellular processes including gene transcription, cell migration, mitochondrial homeostasis, cell proliferation and death [4]. cAMP is synthesized from ATP by adenylyl cyclases, whereas phosphodiesterases (PDEs) hydrolyze this second messenger to its inactive 5′-monophosphate [5].

Currently, 11 human PDE families are known and only PDE4, PDE7 and PDE8 are cAMP-specific. Aberrant expression of PDE induces the imbalance of cAMP levels in the majority of inflammatory cells to promote the several inflammatory disorders, such as chronic obstructive pulmonary disease (COPD) or active psoriatic arthritis [6, 7]. The PDE inhibitors, such as PDE4 inhibitor Roflumilast, are effective anti-inflammatory agents to re-activate the cAMP signals inflammatory processes [6, 8]. Inflammation is the key process in the progression of cancer [9]. However, the function of PDE inhibitors and cAMP signals is elusive during the carcinogenesis. Several evidences indicated that the prospect of targeting PDEs with therapeutic agents for cancer merits consideration. Increased cAMP production in colon cancer was found to inhibit the growth of tumor cells with high levels of PDE3, but not the growth of tumor cells with low levels of PDE3 [10]. Besides, the cAMP analogs, particularly 8-Cl-cAMP, could effectively abrogate the proliferation of medullary thyroid cancer cells and induce apoptosis [11]. Recently, Kelly *et al*. found that PDE4 inhibitor roflumilast and prednisone could restore the cyclic-AMP-mediated growth suppression in B-cell tumors via the inhibition of PI3K/AKT activity [12]. Therefore, we speculated that PDE inhibitor was the potential strategy for the treatment of ovarian cancer via regulation of cAMP signals.

Mitochondrial ferritin (FtMt) is a novel iron-storage protein in mitochondria and encoded by an intronless gene mapped to chromosome 5q23.1 [13]. Santambrogio *et al*. found that FtMt expression was restricted to the cell types characterized by high metabolic activity and oxygen consumption [14]. Interestingly, the hallmarks of tumor contain dysregulation of metabolic activity and oxygen consumption [15], but the role of FtMt in cancer is totally unknown in ovarian cancer. Shi *et al*. demonstrated for the first time a new role and mechanism for FtMt in the regulation of cell cycle. FtMt overexpression disturbed the iron homeostasis of neuronal tumor cell and significantly downregulated the expression of proliferating cell nuclear antigen [16]. In this study, we used PDE4 inhibitor roflumilast to investigate the anti-tumor role for ovarian cancer. The potential mechanisms were analyzed and found that roflumilast activated cAMP/PKA/CERB signals to up-regulate FtMt expression for inhibiting the development of ovarian cancer.

## RESULTS

### Roflumilast inhibits the proliferation and induces apoptosis of ovarian cancer cells *in vitro*

**Figure 1:**
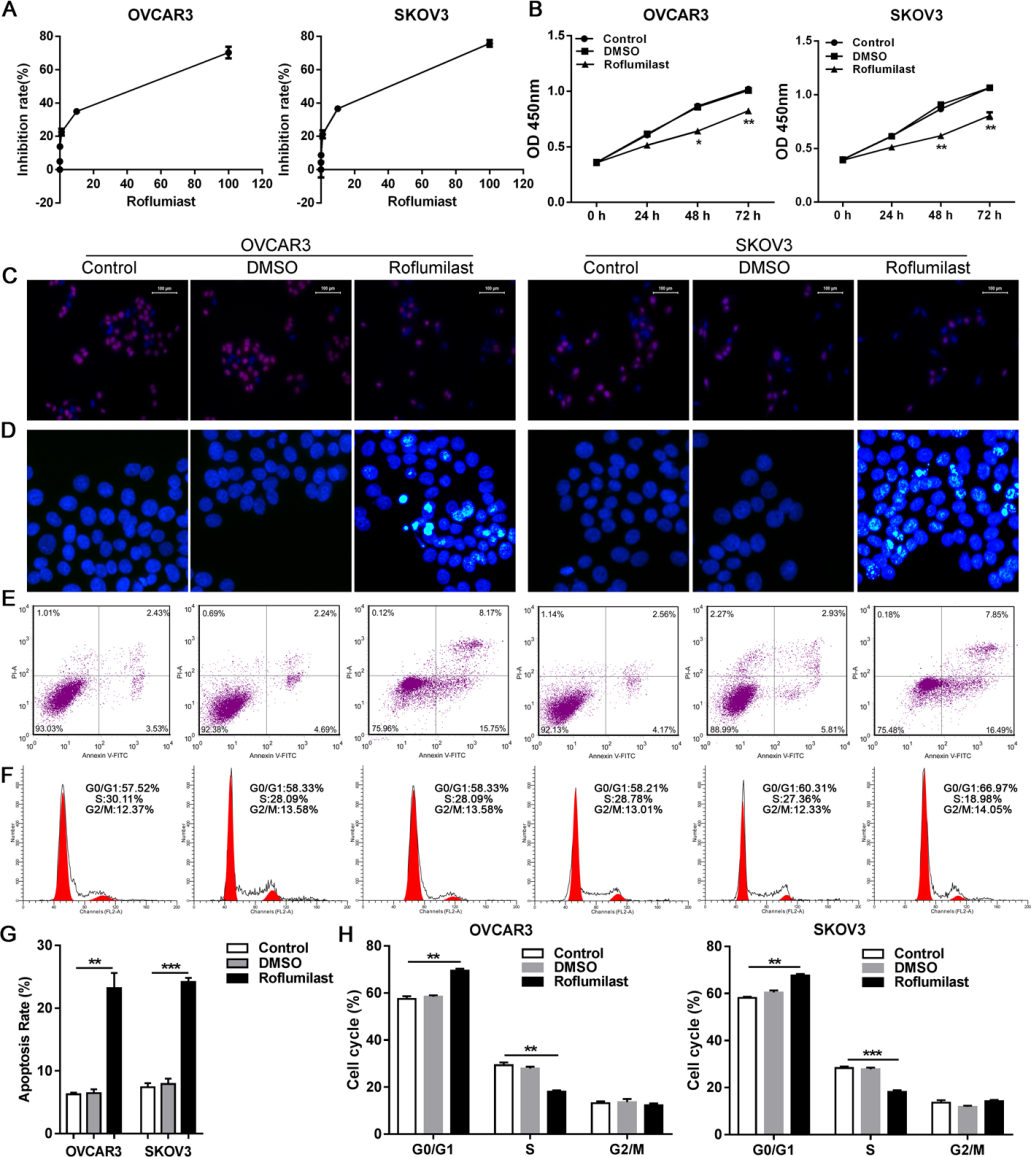
Roflumilast induces cell apoptosis and cell cycle arrest in ovarian cancer (**A**) Different dose of Roflumilast was administrated to OVCAR3 and SKOV3 cells for 24 h and the cell vitalities were determined by CCK-8. (**B**) Two cell lines were treated with Roflumilast (15 μM) for indicated time and the cell vitalities were determined. (**C**) After the treatment of Roflumilast (15 μM) for 48 h, the proliferation and (**D**) cell cycle was analyzed by BrdU assay (magnification: 200x) and flow cytometry. The apoptosis was analyzed by (**E, G**) Hochest assay (magnification: 200x) and (**F, H**) flow cytometry. ^*^p < 0.05, ^**^p < 0.01, data represents the means ± s.d. Representative data are shown from 3 independent experiments.

To uncover the direct anti-tumor effects of Roflumilast on ovarian cancer, two cell lines of ovarian cancer OVCAR3 and SKOV3 were used. A series of concentration of Roflumilast was administrated to two cell lines for 24 h, the results from CCK-8 indicated that Roflumilast significantly inhibit the cell vitality in dose-dependent manner (Figure 1A). Meanwhile, two cell lines were also treated with Roflumilast (15 μM) for indicated time and we found the Roflumilast-induced decreased cell vitality was time-dependent (Figure 1B). BrdU proliferation assay indicated that the proliferation of OVCAR3 and SKOV3 were remarkably reduced by Roflumilast for 48h (Figure 1C). Then, the cell cycle was assessed and the results showed that Roflumilast induced G0/G1 arrest in two cell lines (Figure 1D). Considering that Roflumilast was able to inhibit cell vitality to induce cell cycle arrest, the apoptosis in OVCAR3 and SKOV3 cells was determined (Figure 1E–1G). Significant apoptosis was induced by the treatment of Roflumilast. These data indicated that Roflumilast is an effective drug for the treatment of ovarian cancer.

### Roflumilast activates cAMP/PKA/CREB signals and induces the anti-tumor effects of FtMt

**Figure 2:**
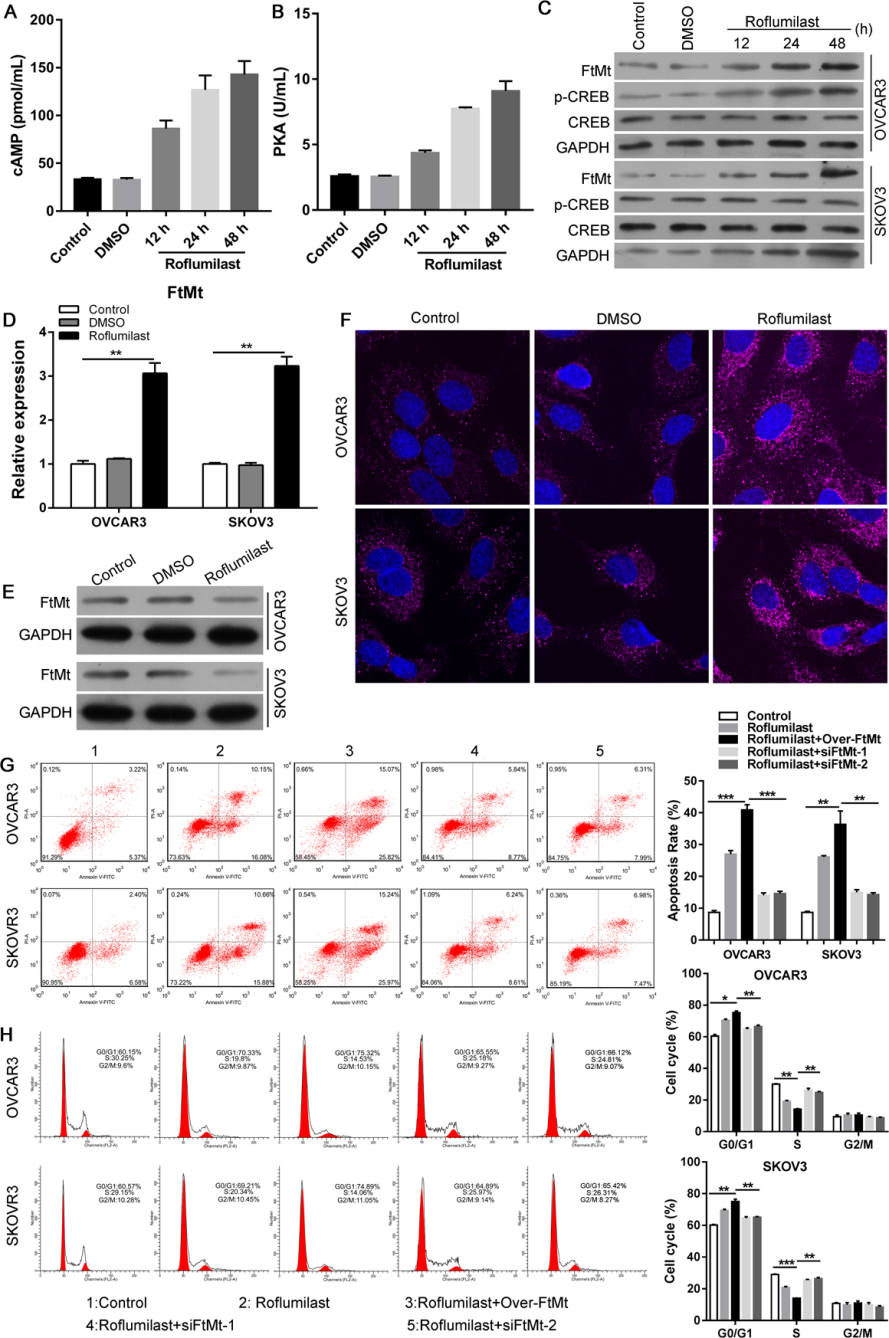
Roflumilast activates cAMP/PKA/CREB signals and induces the FtMt expression for tumor inhibition After the treatment of Roflumilast (15 μM) for 48 h, (**A**) intracellular cAMP levels in cell lysates were measured via the cAMP ELISA kit. (**B**) The activity of PKA was determined by Colorimetric Activity Kit. (**C**) The expressions of CREB and p-CREB in were analyzed by WB. (**D**) The mRNA and (**E, F**) protein level of FtMt were assessed by Q-PCR and WB or immunofluorescence (magnification: 180x). (**G**) Overexpression or (**H**) knockdown of FtMt in Roflumilast-treated OVCAR3 and SKOV3 cells, and the cell apoptosis and cell cycle were analyzed by flow cytometry. ^**^p < 0.01, ^***^p < 0.001, data represent the means ± s.d. Representative data are shown from 3 independent experiments.

We next to investigate the molecular pathways involved into Roflumilast-induced inhibition of tumor. Because Roflumilast is the inhibitor of PDE4, which could hydrolyze and inactivate cAMP, we analyzed the cAMP levels and its downstream signals. The levels of cAMP in SKOV3 cells were elevated in response to the treatment of Roflumilast (Figure 2A), and the activity of cAMP effector PKA showed the similar result that is enhanced by Roflumilast (Figure 2B). In addition, the Roflumilast also promoted the phosphorylation of CREB, suggesting that the cAMP/PKA/CREB pathway was activated by Roflumilast (Figure 2C). Of note, we analyzed the expression of FtMt and found that Roflumilast increased the FtMt expression in two cell lines (Figure 2D–2F). Interestingly, forced expression of FtMt enhanced the Roflumilast-related cell apoptosis (Figure 2G) and G0/G1 arrest (Figure 2H), which, however, was impaired by the knockdown of FtMt. These findings suggested that FtMt could be involved into Roflumilast-induced apoptosis or cell cycle arrest.

### PKA/CREB is required for FtMt-mediated anti-tumor effects of Roflumilast

**Figure 3:**
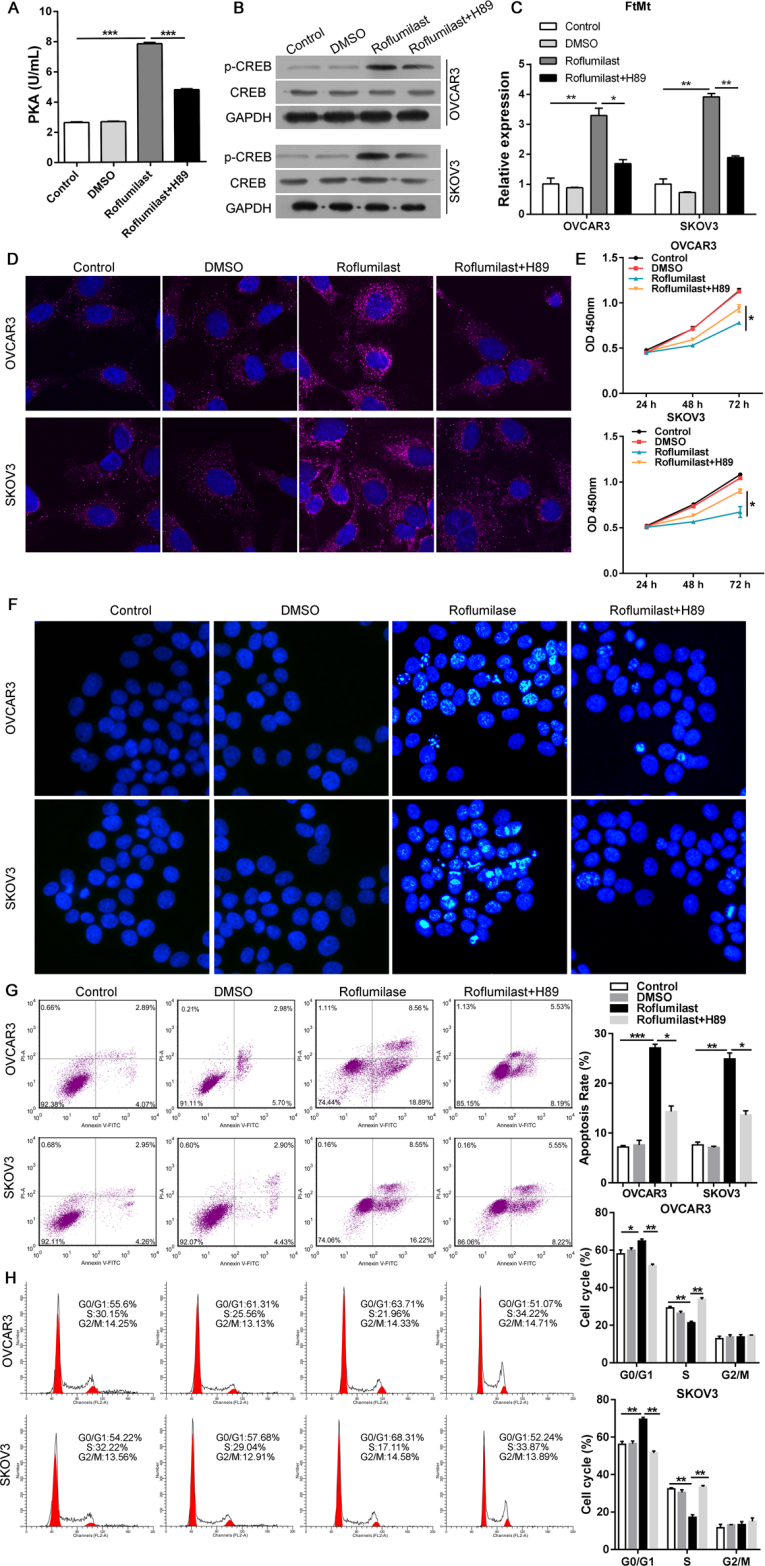
PKA is required for FtMt-mediated anti-tumor effects of Roflumilast After the treatment of H89 (10 μM), the Roflumilast-induced (**A**) PKA activity was analyzed by Colorimetric Activity Kit, and (**B**) the CREB and (**C, D**) FtMt levels were determined by WB, Q-PCR or immunofluorescence (magnification: 180x). (**E**) The cell vitality was analyzed by CCK-8. (**F–H**) The apoptosis and cell cycle were measured by Hochest and flow cytometry. ^*^p < 0.05, ^**^p < 0.01, ^***^p < 0.001, data represent the means ± s.d. Representative data are shown from 3 independent experiments.

**Figure 4:**
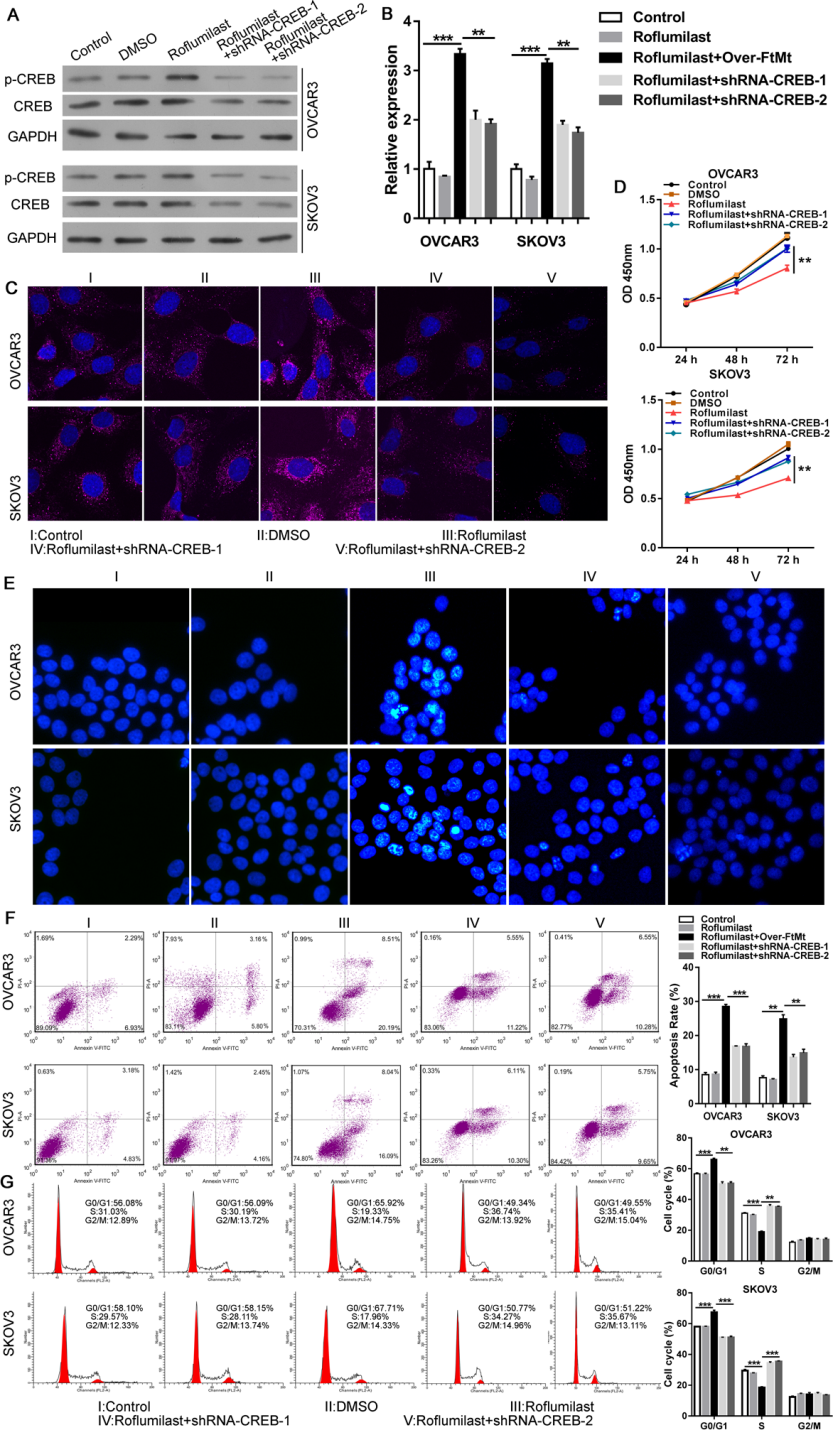
CREB is also required for FtMt-mediated anti-tumor effects of Roflumilast After the knockdown of CREB in OVCAR3 and SKOV3 cells, the Roflumilast-induced (**A**) CREB and (**B and C**) FtMt levels were determined by WB, Q-PCR or immunofluorescence (magnification: 180X). (**D**) The cell vitality was analyzed by CCK-8. (**E–G**) The apoptosis and cell cycle were measured by Hochest and flow cytometry. ^*^p < 0.05, ^**^p < 0.01, ^***^p < 0.001, data represent the means ± s.d. Representative data are shown from 3 independent experiments.

We then analyzed the relationship between cAMP/PKA/CREB and the up-regulation of FtMt in ovarian cancer. The PKA inhibitor H89 was used combined with the Roflumilast (Figure 3A) and the results showed that H89 could inhibit Roflumilast-induced CREB activation (Figure 3B) and the expression of FtMt (Figure 3C and 3D). The proliferation (Figure 3E) and apoptosis assay (Figure 3F–3H) demonstrated that inhibition of PKA was able to abrogate the tumor-killing effects of Roflumilast via down-regulating the expression of FtMt in OVCAR3 and SKOV3 cells. Similarly, when we knockdown the CREB levels in two cell lines (Figure 4A), the expression of FtMt was remarkably decreased (Figure 4B and 4C), which could restore the anti-tumor effects of Roflumilast, including cell vitality, apoptosis and cell cycle (Figure 4D–4G). These data demonstrated that Roflumilast activated cAMP/PKA/CREB signals to promote the FtMt expression, leading to the tumor inhibition.

### CREB directly promotes the transcription of FtMt

**Figure 5:**
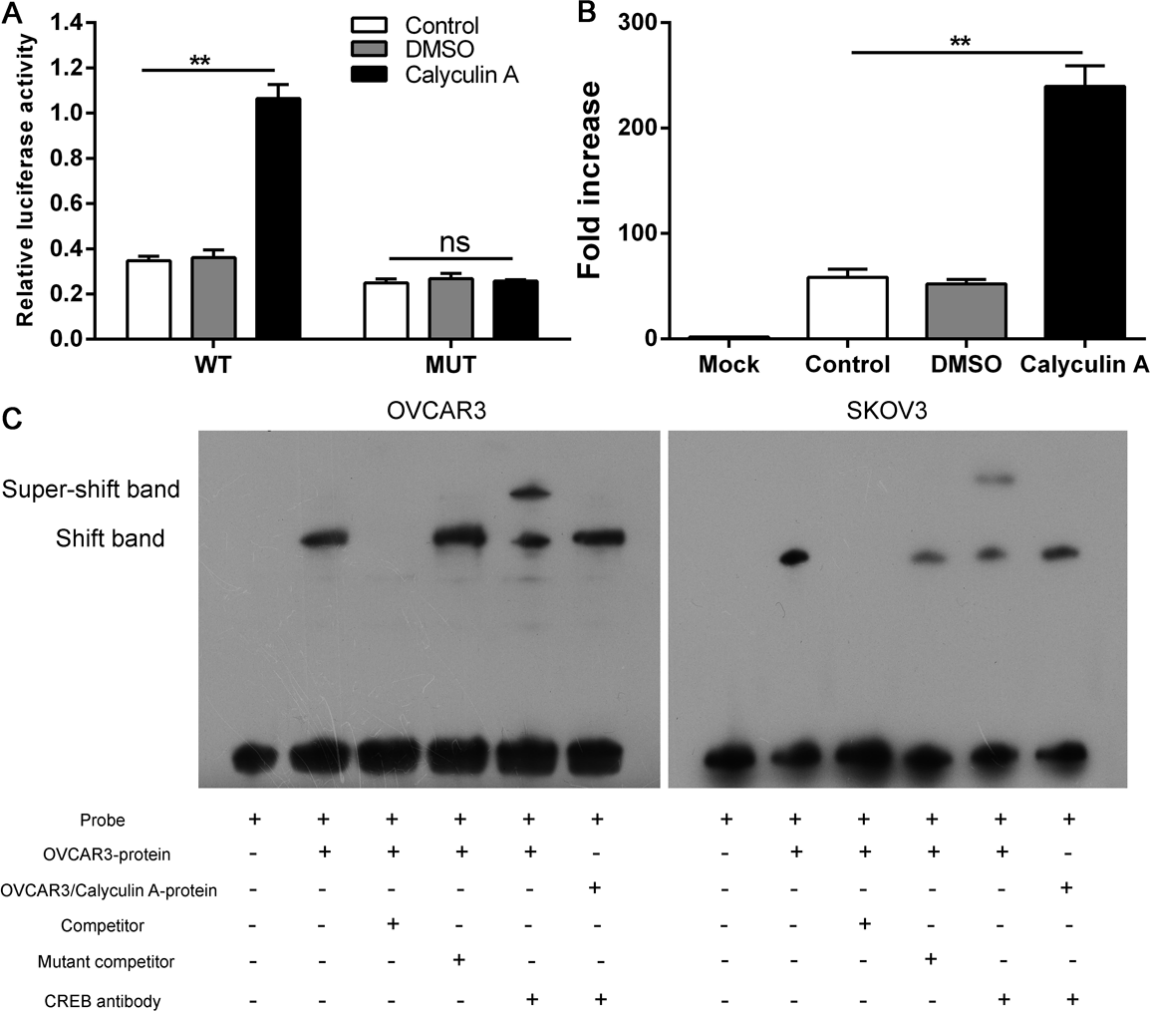
CREB directly promotes the transcription of FtMt (**A**) A luciferase reporter vector with full-length promoter construct of FtMt containing mutation at CREB binding sites was transfected into SKOV3 cells and the luciferase activity were determined. (**B**) SKOV3 cells were subjected to ChIP assay with anti-CREB antibody, The PCR product was detected in cell nuclear extracts (input) as well as in SKOV3 cells, which was confirmed by (**C**) EMSA, ^32^P-labeled oligonucleotide probes corresponding to the FtMt promoter, complexed with CREB in the presence or absence of anti-CREB antibody or specific/mutant competitors were detected by EMSA. ^**^p < 0.01, data represent the means ± s.d. Representative data are shown from 3 independent experiments.

We found that Roflumilast could phosphorylate CREB to up-regulate the level of FtMt, and the promoter region of FtMt was reported to harbor the binding site of CREB [17], which implicated that the increased expression of FtMt in ovarian cancer was attributed to the transcription of CREB. To confirm the binding site of CREB within FtMt promoter region in ovarian cancer, the luciferase reporter assays were performed by using Calyculin A which was demonstrated to be the activator of CREB. A luciferase reporter vector with full-length promoter construct of FtMt containing mutation at CREB binding sites was transfected into SKOV3 cells and the results showed that the luciferase activity of wild-type promoter construct of FtMt could be enhanced by Calyculin A, which, however, was negative in two mutant-types promoter construct of FtMt (Figure 5A). We further carried out a ChIP with anti-CREB antibody, and oligonucleotide probes with mutations of the FtMt motif sites were synthesized for EMSA analysis. The results confirmed that CREB directly binds to FtMt promoter to enhance its transcription activation in OVCAR3 and SKOV3 cells (Figure 5B and 5C).

### Roflumilast activates cAMP/PKA/CREB/FtMt to restrict the growth of ovarian cancer *in vivo*

**Figure 6:**
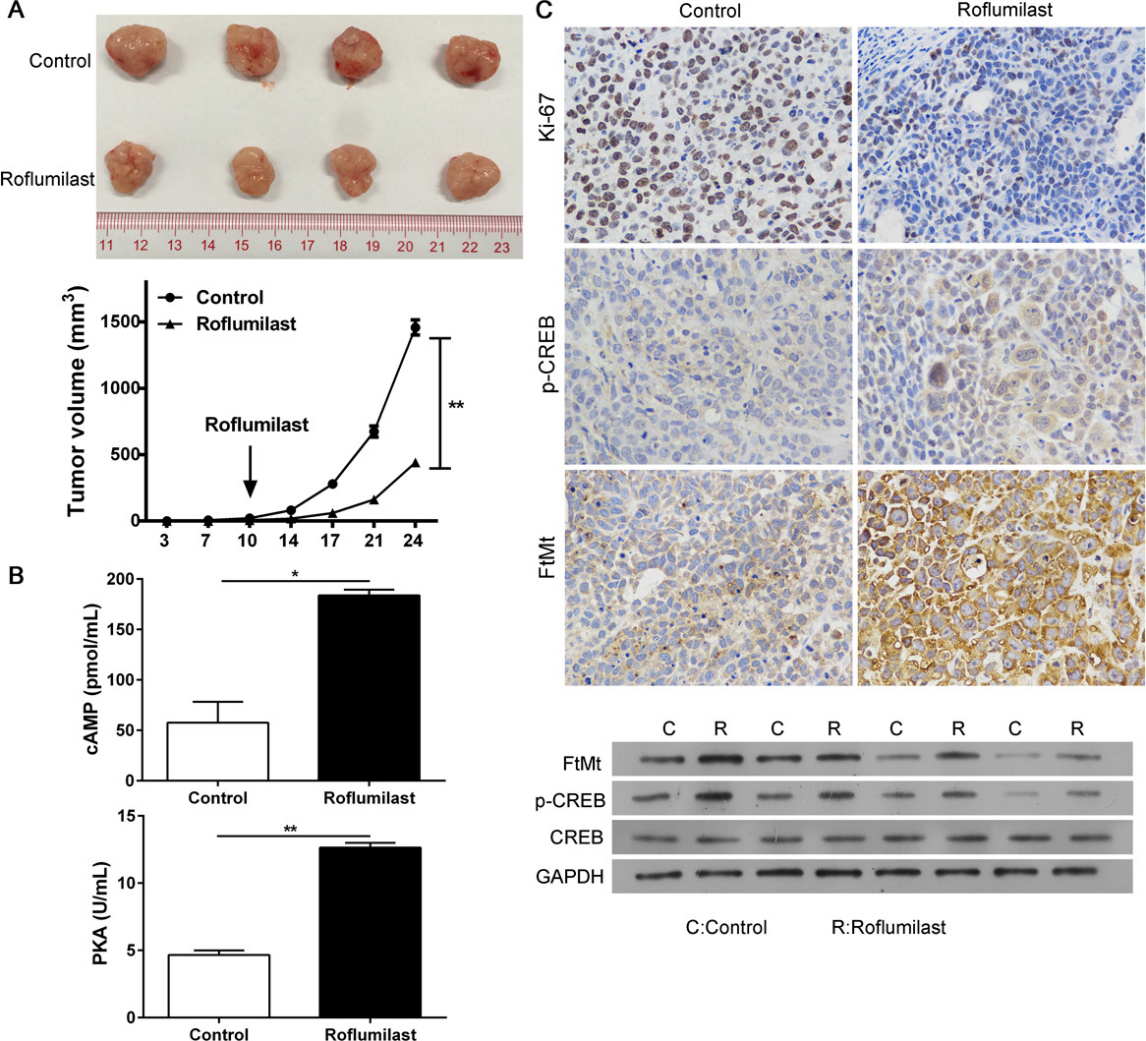
The anti-tumor effects of Roflumilast *in vivo* 2 × 10^6^ SKOV3 cells were subcutaneously injected in rear flank of nude mice (4 per group) and 5 mg/kg Roflumilast or PBS was administrated (i.p.) to mice. (**A**) The mean tumor size (mm3) was analyzed. (**B**) The cAMP levels and PKC activity were estimated. (**C**) The expressions of Ki-67, p-CREB and FtMt were determined by immunohistochemistry and WB (magnification: 200×). ^*^p < 0.05, Data represent the means ± s.d. ^*^p < 0.05, ^**^p < 0.01, data represent the means ± s.d.

To provide the evidence of anti-tumor effects of Roflumilast *in vivo*, the xenograft model of SKOV3 cells were established and the nude mice were treated with Roflumilast (5 mg/kg i.p.). The results showed that the administration of Roflumilast could effectively inhibit tumor growth and restrict the tumor volume (Figure 6A). The tumor proliferation was determined by Ki-67 staining via IHC and we found that the treatment of Roflumilast inhibited the level of Ki-67. Moreover, the cAMP signals and the FtMt levels were also confirmed *in vivo*. The level of cAMP and activities of PKA in mice treated with Roflumilast was significantly higher than that in control groups. The phosphorylated CREB and FtMt expressions were also induced by Roflumilast *in vivo* (Figure 6B and 6C).

## DISCUSSION

Due to the presentation at late stage, only 15% patients are diagnosed with local disease and the overall survival rate of five years remains only about 30% in ovarian cancer [18]. For the majority of patients with advanced ovarian cancer, debulking surgery and platinum-based chemotherapy are the common strategy of treatment, but the response rate is low [3]. Eventually, once the patients develop recurrent disease, standard therapies will be unsuitable for those patients. Thus, the discovery of alternative treatment is indispensable for new treatment of ovarian cancer. Here, we uncovered the anti-tumor effects of PDE4 inhibitor Roflumilast for ovarian cancer via activating PKA/CREB/FtMt pathway.

Currently, selective PDE inhibitors have been investigated for the treatment of a wide range of diseases, such as chronic obstructive pulmonary disease (COPD), asthma, pulmonary hypertension and erectile dysfunction etc [19]. PDE2 inhibitor was found to be of therapeutic interest in sepsis and acute respiratory distress syndrome. The selective PDE4D inhibitor, GEBR-7b, was reported to improve memory in rodents at non-emetic doses [7, 20]. However, notably absent from these efforts is a detailed examination of PDE inhibitors in cancer therapeutics. PDE4 inhibitors Roflumilast could up-regulate glucocorticoid receptor (GRa) transcript levels in B Cell Chronic Lymphocytic Leukemia (B-CLL) cells but not T-CLL cells to enhance the glucocorticoid-mediated apoptosis [21]. We here demonstrated that the administration of Roflumilast functioned as an anti-tumor therapeutics for ovarian cancer via inducing apoptosis and inhibiting proliferation of cancer cells. Similarly, Tagawa T *et al*. reported that PDE1C mRNA is overexpressed in human malignant melanoma-associated antigen (MAA) cells, and growth is inhibited by the PDE1 inhibitor vinpocetine [22]. But they also found that the PDE3-specific inhibitors did not inhibit the proliferation of MAA cells, despite of the presence of high PDE3 activity [23]. Besides, Dong *et al*. reported that the PDE inhibitor of PDE3, PDE4, PDE7 and PDE8 impaired the migration of aggressive triple negative MDA-MB-231 breast cancer cells but had little effect on breast cancer cell proliferation [24], which implicated the inconsistence between PDE activity and anti-tumor effects in different cancer.

The mechanisms involved in the treatment of PDE inhibitors have not been known clearly. Kelly *et al*. found that roflumilast and prednisone could restore the cAMP-mediated growth suppression in B-cell tumors via the inhibition of PI3K/AKT activity [12]. In addition, the anti-angiogenesis role of Roflumilast in diffuse large B-cell lymphoma (DLBCL) was also reported, PDE4B repressed cAMP to activate PI3K/AKT signals to up-modulate VEGF secretion and Roflumilast could decreased the microvessel density in lymphoma-bearing mice [25]. In ovarian cancer, we found that Roflumilast restored the cAMP level to promote PKA/CREB signals and inhibit tumor growth. However, increased cAMP also initiated protective effects for cell survival. Zhang *et al* found that cAMP-mediated inhibition of JNK activation antagonized UV-induced apoptosis in CREB-dependent manner [26]. Activation of cAMP/PKA/CREB signaling axis in cancer-associated fibroblasts triggered the aerobic glycolysis to provide extra pyruvate and lactate to tumor cells for multidrug resistance [27]. Therefore, the function of PDE/cAMP/CREB signals in cancer is dependent on the discrepancy of downstream target genes.

Evidences have identified the role of FtMt in protecting mitochondria from iron-dependent oxidative damage via sequestration of potentially harmful excess free iron [13]. To be noted, the expression pattern of FtMt was associated with the hallmarks of cancer that highly oxidative and metabolic activity, suggesting that it might participate into tumorigenesis. Indeed, FtMt was found to dramatically inhibit human neuroblastoma cells SH-SY5Y proliferation and tumor growth in nude mice [16]. In this study, CREB was demonstrated to directly promote the transcription of FtMt, and the increased expression of FtMt inhibited the proliferation and induced G0/G1 arrest of ovarian cancer cells. These findings indicated an anti-tumor role of FtMt.

In conclusion, the treatment of Roflumilast is able to re-activate cAMP/PKA/CREB signaling axis in ovarian cancer, leading to FtMt-mediated tumor inhibition *in vitro* and *vivo*. Therefore, the application of PDE inhibitors for cancer merits consideration in clinical.

## MATERIALS AND METHODS

### Cell lines and reagents

The ovarian cancer cell line OVCAR3 and SKOV3 were purchased from the cell bank of the Chinese academy of sciences (Shanghai, China) and cultured in Dulbecco's Modified Eagle's medium (DMEM) supplemented with 10% FBS (Life Technologies, USA), ampicillin and streptomycin at 37°C, 5% CO2 conditions. PDE4 inhibitor Roflumilast and PKA inhibitor H89 and Calyculin A were purchased from Sigmae Aldrich (St. Louis, MO, USA). Anti-GAPDH, CREB, p-CREB and FtMt antibodies were obtained from Cell Signaling Tech (Denver, MA, USA) and Abcam (USA). For overexpression of FtMt or knockdown of CREB, the overexpression vector pcDNA3.0-FtMt or shRNA-CREB were conducted by Vigene Biosciences, CH801187 (China) and GenePharma (Shanghai, China). Reporter plasmid of full-length promoter construct (wild-type or mutant) of pGL-3-FtMt was conducted by GenePharma (Shanghai, China).

### CCK-8 assay

After the treatment of Roflumilast in different dose to OVCAR3 and SKOV3 cell lines for indicated time, the cells were harvested and washed with PBS and then cell counting kit-8 (Kumamoto, Japan) mixed with DMEM was used for cell viability assay, and the absorbance was measured at 450 nm by a microplate reader.

### Flow cytometry assay

For the BrdU assay, the OVCAR3 and SKOV3 cells were treated with Roflumilast (15 μM) for 48 h, and then the cells were harvested and suspended with PBS, then cells were stained by for BrdU incorporation 8 hours after all experimental conditions with the BrdU Labeling and Detection Kit I (Roche). Cells pre-labeled with BrdU were fixed with ethanol and then incubated with monoclonal antibodies against BrdU mixed with nucleases, followed by fluorescein-conjugated secondary antibodies and quantified by flow cytometry on a FACS Calibur instrument. For the cell apoptosis, 2 μl of annexin V mixed with 2 μl of Propidium iodide (PI, eBioscience) were used to stain dispersed cell suspensions cells, 10000 cells were collected for the analysis of flow cytometry. For the cell cycle analysis, cells were stained with PI staining solution (10 μg/ml RNase A and 50 μg/ml PI) at 37°C for 30 min in dark, 60000 cells were collected for the analysis of flow cytometry. The cell cycle distribution was analyzed using a flow cytometry provided with the Cell-Quest software.

### Hoechst staining assay

OVCAR3 and SKOV3 cells treated with Roflumilast (15 μM) for 48 hours, and then were washed by PBS and fixed by PFA. The cells stained with 0.1 μg/ml Hoechst 33342 (Sigma, St Louis, MO, USA); Fluorescence microscopy with a filter for Hoechst 33342 (365 nm) was used to detected the changes of nuclear morphology.

### cAMP levels and PKA activity measurements

Intracellular cAMP was determined in two cell lines using the cAMP competitive ELISA kit purchased from Invitrogen (USA, Catalog number: EMSCAMPL). The PKA Colorimetric Activity Kit (USA, Catalog Number: EIAPKA) was used to measure PKA activity. Samples of cell lysates were prepared exactly as described by the manufacturer.

### Western blots

OVCAR3 and SKOV3 cells transfected with shRNA-CREB or negative control were treated with Roflumilast and/or H89, and then the cells were harvested and, according to the manufacturer's instructions, the whole cell protein extracts were resolved on a 10% SDS denatured polyacrylamide gel and were then transferred onto a nitrocellulose membrane, which were blocked in 5% BSA in TBST buffer (Tris Buffer Saline containing 0.1% Tween-20) for 1 h at room temperature, and subsequently incubated with Anti-GAPDH (#ab8245, Abcam, USA), FtMt (#ab124889, Abcam, USA), CREB (#ab32096, Abcam, USA), p-CREB (#9198, Cell Signaling Technology, USA) antibodies overnight at 4°C. GAPDH was used as the loading control in the Western blotting. After washing with TBST buffer, the blots were then incubated with HRP-conjugated secondary antibody (#BS12478, #BS13278, Bioworld, China) for 1 h at room temperature. After washing with TBST buffer, the blots were visualized using the ECL-Plus reagent (Millipore, Billerica, MA, USA).

### RNA isolation and qRT-PCR

OVCAR3 and SKOV3 cells transfected with shRNA-CREB or negative control were treated with Roflumilast and/or H89, and then the cells were harvested, PBS was used to wash for three times, total RNA from OVCAR3 and SKOV3 cells was extracted using Trizol reagent (Invitrogen) according to the standard RNA isolation protocol. Quantitative real-time RT-PCR (qRT-PCR) was performed, and the expression levels of FTMT were normalized to GAPDH for gene expression.

**Table:** 

Gene	Primer
GAPDH-F	ACACCCACTCCTCCACCTTT
GAPDH-R	TTACTCCTTGGAGGCCATGT
FTMT -F	TGGAGTGTGCTCTACTCTTGG
FTMT -R	ACGTGGTCACCTAGTTCTTTGA

### Cell transfection

To knockdown the CREB, the OVCAR3 and SKOV3 cells were seeded into 12 plate and transfected with shRNA pGenesil-CERB or negative control for indicated time at a concentration of 1 ng/mL by Lipofectamine 2000 (Invitrogen, USA) according to the manufacturer's instructions.

### Dual-luciferase assay

To investigate the direct target of CREB on the transcription of FtMt, the SKOV3 cells were co-transfected containing 200 ng firefly Luciferase vector, 40 ng Renilla luciferase pRL-TK vector (Promega, USA) and pGL-3-promoter construct of FtMt (wild-type or mutant). Luciferase activity was measured using the Dual-Luciferase Reporter Assay System (Promega, USA). Firefly luciferase acted as a reporter gene and Renilla luciferase as a normalized control.

### ChIP assay

For ChIP assay, SKOV3 cells were then lysed and chromatin was harvested to fragmented and then subjected to immunoprecipitation using antibodies specific to CREB (ab31387). Protein

G-Dynabeads were washed in PBS, 0.5% BSA O/N at 4°C. IP samples were added to Dynabeads and incubated for 2 h at 4°C rotating. The beads were then washed 3 times for 10 min each at 4°C rotating. After the last wash, the supernatant was removed, 120 μl of decrosslink solution (100 mM NaHCO3, 1% SDS) was added in each sample and incubated O/N at 65°C with shaking. The protein/DNA cross-links were reversed and the DNA was purified. The DNA sequences interacted with protein of CREB were co-precipitated as part of the cross-linked chromatin complex and these DNA sequences was determined by quantitative real time PCR using specific primers of FtMt.

**Table:** 

Gene	Primer
FTMT-wild-F	GGGGTACCTACACTTGCTTTGGATGTGGACCTTTAT KpnI
FTMT-wild-R	CCGCTCGAGAACTGAGGCCCGGCCCTCTT XhoI
FTMT-MUT-F	CGTTCTGATCAGATCCATTCGGACCAGCGCCCGACCCC
FTMT-MUT-R	GGGGTCGGGCGCTGGTCCGAATGGATCTGATCAGAACG

### Electrophoretic mobility shift assay (EMSA)

According to the manufacturer's instructions, OVCAR3 and SKOV3 cells treated with Calyculin A for 48 hours, and the nuclear extracts was prepared. The DNA-binding activity of CREB was determined by EMSA using nuclear extracts prepared by a nuclear extract kit (Active Motif; Rixensart, Belgium) from different groups of cells. The related probes were synthesized (Invitrogen) and incubated with nuclear extracts in the reaction solution. Unlabeled wild-type (site: GCCCCCTTTCCCCCAGGGCTGAAGGGACC) and mutant (site: GCCCCCTTAGGGGGTCCCGAGAA GGGACC) double-stranded competitor oligonucleotides were added to the respective reactions. The specificity of binding was examined by competition with the unlabeled self, mutant and consensus oligonucleotides.

### Tumor model

To investigate the anti-tumor effects of Roflumilast *in vivo*, 2 × 10^6^ SKOV3 cells were subcutaneously injected in rear flank of nude mice (4 per group) and 5 mg/kg Roflumilast or PBS was administrated (i.p.) to mice for five times, three days apart. Tumor diameters were measured with a digital caliper every other day and the tumor volume in mm^3^ was calculated as volume = π/6(width)^2^ × length.

### Immunohistochemistry

The expressions of ki-67, p-CREB and FtMt in ovarian cancer tissues were analyzed by IHC and 2-μm-thick, formalin-fixed and paraffin-embedded specimen sections were used. After the slides were incubated in xylene for 5 min, 100% ethanol was used for 10 min, 95% ethanol for 10 min. Antigen unmasking was performed and then the slides were blocked with 3% hydrogen peroxide for 30 min at room temperature. Then the primary antibody for ki-67, p-CREB and FtMt was incubated the FFPE specimen sections at 4°C overnight and then the biotinylated horse second antibody and streptavidin-horse-radish peroxidase (Zymed Laboratories Inc.) were used for the detection of ki-67, p-CREB and FtMt. After that, the EnVision Detection System kit (DAKO, Denmark) was used for the DAB chromogen followed by nuclear staining using haematoxylin.

### Immunofluorescence

Cells were fixed with 4% formaldehyde in PBS for 15 min and rinsed with PBS for three times. Then cells were permeabilized with 100% methanol for 10 min at −20°C and blocked with 3% bovine serum albumin (BSA) in PBS for 60 min and incubated with primary antibodies for FtMt (#ab66111, Abcam, USA) overnight at 4°C. After rinsing three times in PBS, incubated coverslips in fluorochrome-conjugated secondary antibody for 1–2 h at room temperature in dark and then stained the nucleus with DAPI. The coverslips were mounted onto the glass slides with neutral gum and observed by FV10i confocal microscope (OLYMPUS, Japan).

### Statistical analysis

All statistical analyses were performed by Statistical Program for Social Sciences (SPSS) 16.0 software (SPSS, Chicago, IL, USA) and GraphPad Prism 5.0 (GraphPad Software, La Jolla, CA, USA). Unpaired *t*-tests or Mann–Whitney *U* tests were used to compare the two groups, and multiple group comparisons were analyzed with one-way ANOVA. *P* < 0.05 was considered to be statistically significant.

## Abbreviations

cAMP: cyclic adenosine monophosphate
PKA: protein kinase A
CREB: cyclic AMP response element-binding protein
PDEs: phosphodiesterases
FtMt: mitochondrial ferritin
COPD: chronic obstructive pulmonary disease
DMEM: Dulbecco's Modified Eagle's medium
PI: propidium iodide
EMSA: electrophoretic mobility shift assay
GRa: glucocorticoid receptor
BCLL: B cell chronic lymphocytic leukemia
MAA: malignant melanoma-associated antigen
DLBCL: diffuse large B-cell lymphoma
